# Addressing High-Grade Pivot Shift: Clinical Outcomes of Internal Brace-Augmented Anterior Cruciate Ligament (ACL) Reconstruction

**DOI:** 10.7759/cureus.111241

**Published:** 2026-06-21

**Authors:** Naveen Gowda, Bhagwati Prasad Sharma, Amit M Hegde, Chandra Kant Singh, Nilesh Verma, Harshita Harshita, Abhishek Chakraborty, Praveen Shetty

**Affiliations:** 1 Orthopedics, Vardhman Mahavir Medical College and Safdarjung Hospital, New Delhi, IND; 2 Orthopedics, Safdarjung Hospital, New Delhi, IND

**Keywords:** acl reconstruction, anterior cruciate ligament, arthroscopic reconstruction, fibertape, functional outcomes, hamstring autograft, internal brace augmentation, knee stability, pivot shift, rotational instability

## Abstract

Background and objective

Anterior cruciate ligament (ACL) injuries associated with high-grade pivot shift represent a challenging subset of rotational knee instability. Internal brace augmentation using FiberTape (Arthrex, Inc., Naples, FL, USA) has been proposed to enhance graft protection during early healing and rehabilitation following ACL reconstruction. The objective of this study was to evaluate the functional and clinical outcomes of arthroscopic ACL reconstruction with internal brace augmentation in patients presenting with Grade III preoperative pivot shift.

Methods

A prospective cohort study was conducted at a tertiary care center involving patients with complete ACL tears and Grade III pivot shift who underwent arthroscopic ACL reconstruction with hamstring autograft and FiberTape augmentation. Patients with multiligament injuries, revision surgery, generalized laxity, or significant meniscal injuries requiring extensive intervention were excluded. Functional outcomes were assessed using the Knee Injury and Osteoarthritis Outcome Score (KOOS), Lysholm Knee Score, and International Knee Documentation Committee (IKDC) score at predefined follow-up intervals up to 12 months. Clinical stability was evaluated using Lachman and pivot shift tests.

Results

A total of 43 patients completed the study. Progressive and statistically significant improvement was observed across all KOOS domains, Lysholm scores, and IKDC scores throughout follow-up. By 12 months, most patients achieved excellent functional outcomes, with marked improvement in sports activity and quality-of-life measures. Clinical examination demonstrated restoration of rotational stability in the majority of patients, with most achieving Grade 0 pivot shift and Lachman test results at final follow-up. No patient demonstrated high-grade residual laxity during the study period.

Conclusions

Arthroscopic ACL reconstruction with internal brace augmentation demonstrated excellent functional recovery and restoration of rotational stability in patients with Grade III pivot shift. The technique appears to be a valuable adjunct in managing severe rotational instability, with favorable short-term clinical outcomes and stable knee function during follow-up.

## Introduction

Anterior cruciate ligament (ACL) injuries are among the most disabling musculoskeletal injuries in orthopedics, with over 200,000 cases each year in the United States and a particularly high rate among young, active individuals [1]. The ACL primarily prevents anterior translation of the tibia and helps control knee rotation [2]. It is subjected to the greatest stress during cutting, pivoting, and deceleration movements, which are common in sports such as football, basketball, and gymnastics. This makes the ACL especially prone to injury in both contact and noncontact situations [3]. Most injuries occur without contact, usually during sudden deceleration, valgus stress, and tibial rotation [4,5].

If an ACL injury is not treated, it can lead to long-term problems such as persistent instability, recurrent meniscal injuries, cartilage damage, and early osteoarthritis, which may appear within 10 years of the initial injury [6,7]. The pivot shift test is especially important for assessing ACL injuries because it reproduces the sensation of the knee giving way and evaluates knee instability during dynamic movement [8]. A high-grade pivot shift, such as Grade II or III, indicates severe rotational laxity and is often associated with injury to the anterolateral capsule or anterolateral ligament, which contributes to control of tibial rotation [9].

Arthroscopic ACL reconstruction is the standard surgical procedure for restoring knee stability in active patients [10]. The most commonly used grafts include quadruple hamstring autografts using the semitendinosus tendon, sometimes combined with the gracilis tendon, and bone-patellar-tendon-bone grafts [11,12]. Despite advances in surgical techniques, some patients still experience graft failure or persistent rotational instability, with rerupture rates ranging from 8% to 17% in recent studies [13]. The graft is particularly vulnerable during the months required for revascularization and ligamentization, when it may stretch or fail under physiological loads.

To address this issue, surgeons have introduced internal brace augmentation using high-strength suture tape, most commonly FiberTape (Arthrex, Inc., Naples, FL, USA; ultrahigh-molecular-weight polyethylene), as an adjunct to standard reconstruction [14,15]. The internal brace acts as a secondary stabilizer, sharing load with the graft during the critical healing period. This limits elongation and protects the reconstructed ligament from excessive stress [16]. This approach increases construct stiffness and strength without restricting normal joint motion when appropriate tensioning is achieved [16]. These advantages may allow for faster rehabilitation and earlier return to sports, which is particularly relevant for patients with severe preoperative pivot shift.

While research on internal brace augmentation is increasing globally, there remains limited prospective data from India, particularly in patients with Grade III pivot shift. This study aims to prospectively evaluate the functional and clinical outcomes of arthroscopic ACL reconstruction with internal brace (FiberTape) augmentation in patients with preoperative Grade III pivot shift, using validated patient-reported outcome measures over 12 months.

## Materials and methods

Study design and setting

This prospective cohort study was conducted at a single tertiary care center over an 18-month recruitment period. Institutional Ethics Committee approval was obtained prior to commencement, and written informed consent was obtained from all participants. The study was conducted in accordance with the Declaration of Helsinki.

Patient selection criteria

Patients between 18 and 60 years of age with a complete ACL tear confirmed by clinical examination and MRI, as well as a Grade III pivot shift before surgery, were considered for the study. Patients were excluded if they had multiligament injuries, previous surgery on the same knee, generalized ligament laxity, fractures near the joint, active infections, or failed ACL reconstruction.

Of 65 patients enrolled at the time of surgery, 22 were excluded postoperatively based on a criterion predefined in the study protocol: intraoperative finding of significant meniscal injury requiring surgical treatment (partial meniscectomy of more than one-third of the meniscal width or meniscal repair). Patients with minor meniscal pathology, defined as small stable tears not requiring intervention or partial meniscectomy involving less than one-third of the meniscal width, were retained in the analysis, as such findings were not expected to materially influence the accelerated rehabilitation protocol or primary outcome measures. Meniscal injuries necessitating major surgical treatment were excluded because their rehabilitation requirements differ substantially from the accelerated protocol employed in this study, and their inclusion would have introduced a confounding source of outcome variability. The final analyzed cohort comprised 43 patients.

Outcome measures

All patients underwent standard preoperative evaluation, including clinical examination (Lachman test, anterior drawer test, and pivot shift grading), weight-bearing radiographs, and MRI. Baseline knee function was measured using three validated tools: the Knee Injury and Osteoarthritis Outcome Score (KOOS) [17,18], the Lysholm Knee Score [19], and the International Knee Documentation Committee (IKDC) score [20].

Surgical procedure

All procedures were performed under spinal or general anesthesia with patients in the supine position. A padded tourniquet was placed on the proximal thigh, and the knee was positioned at 90° of flexion using a leg holder. Standard anterolateral and anteromedial portals were created, followed by a thorough diagnostic arthroscopy of all compartments. ACL deficiency was confirmed by visualization of an empty femoral notch and residual tibial footprint fibers. If patients had minor meniscal pathology, these were treated with partial meniscectomy or repair as indicated. Patients requiring major meniscal surgery were not included in the final cohort.

Hamstring grafts were harvested through a 2-3 cm oblique incision over the pes anserinus. The semitendinosus and gracilis tendons were identified and separated, ensuring protection of the superficial medial collateral ligament and saphenous nerve branches. The tendons were harvested using an open-ended stripper after complete release of the vinculae. The graft was then folded into a quadruple-strand construct and tensioned on a preparation board. The mean graft diameter in this cohort was 8.5 mm (minimum 8 mm), and the mean graft length was 12 cm (minimum 7 cm), consistent with recommended thresholds for quadrupled hamstring constructs.

The femoral socket was prepared through the anteromedial portal using an inside-out technique at the anatomic footprint. The tibial tunnel was created using a guide aligned with the anterior horn of the lateral meniscus and the native ACL footprint. FiberTape was placed alongside the graft, running parallel without excessive tension, and secured with a femoral cortical button. The graft was passed using shuttle sutures. Fixation was performed in two steps: the hamstring graft was tensioned and fixed at 30° of knee flexion using a tibial bioabsorbable interference screw, followed by separate fixation of the FiberTape with a knotless suture anchor near full extension. This technique allows the graft to assume the primary load, while the FiberTape serves as a secondary stabilizer to protect the graft during early rehabilitation without restricting knee motion. Final arthroscopy confirmed appropriate graft position, absence of notch impingement, and stable knee kinematics.

Postoperative rehabilitation protocol

Patients followed a structured accelerated rehabilitation protocol. Weight-bearing with a walker and a long knee brace was permitted from postoperative day 1. Both the brace and walker were discontinued at two weeks. Full range of motion was targeted by four to six weeks, after which progressive resistance strengthening was initiated. Proprioception and neuromuscular training, including gym-based exercises, were added once full range of motion was achieved. A formal return-to-sport assessment was performed at six months. Return to preinjury sport was permitted only if all three of the following criteria were met: (1) a limb symmetry index (LSI) of at least 90% on single-leg hop for distance and the 6-meter timed hop test; (2) isokinetic quadriceps strength LSI of at least 85% at 60°/s and 180°/s on Biodex dynamometry; and (3) surgeon confirmation of full clinical stability and complete range of motion.

Patients were reviewed at six weeks, three months, six months, and 12 months postoperatively. KOOS [17,18], Lysholm [19], and IKDC [20] scores were reassessed at each visit. Clinical knee stability was evaluated using Lachman and pivot shift tests at the six- and 12-month assessments.

Sample size calculation

The sample size was calculated to detect a clinically meaningful improvement in functional outcome, based on the established minimal clinically important difference (MCID) of approximately 11.5 points for the IKDC score and 8-10 points for KOOS subscales [18,20]. Using an expected mean improvement (Δ) of 12 points, an SD of 15 points from comparable ACL outcome literature, 80% power (Zβ = 0.84), and a two-tailed alpha of 0.05 (Zα/2 = 1.96), the minimum required sample size for a paired comparison was:

\(n = \left[\frac{(Z_{\alpha/2} + Z_{\beta}) \times \sigma}{\Delta}\right]^2 = \left[\frac{(1.96 + 0.84)\times 15}{12}\right]^2 = \left[3.5\right]^2 = 12.25 \approx 13 \text{ patients}\)

Given that this minimum estimate assumed ideal conditions, a larger cohort of 43 patients was enrolled to improve precision of effect estimates, enhance generalizability, and account for protocol deviations, consistent with conservative powering recommendations when a large effect size (Cohen’s d > 0.8) is anticipated in a clinically heterogeneous population.

Statistical analysis

Continuous variables were described as mean ± SD; categorical variables as frequencies and percentages. Friedman’s nonparametric test was used for repeated-measures comparisons across time points, with post hoc Wilcoxon signed-rank tests and Bonferroni correction applied. Effect sizes were quantified using paired Cohen’s d, calculated as the mean difference between preoperative and 12-month scores divided by the preoperative SD. This approach was selected as a conservative paired-design estimate appropriate for the repeated-measures structure of the data, given that individual difference scores were not retained for post hoc computation. Approximate 95% CIs for Cohen’s d were estimated using the standard error formula:

$SE = \sqrt{\left(\frac{1}{n} + \frac{d^2}{2n}\right)}$

yielding

$CI = d \pm 1.96 \times SE$

MCID thresholds applied were 8-10 points for KOOS [18], approximately 10 points for the Lysholm score [19], and approximately 11.5 points for the IKDC score [20]. All analyses were performed using IBM SPSS Statistics for Windows, version 28.0 (released 2021; IBM Corp., Armonk, NY, USA). Statistical significance was defined as p < 0.05 (two-tailed).

## Results

The final study cohort comprised 43 patients. The mean age was 26.9 ± 5.8 years (median 25; range, 19-42 years). The cohort was predominantly male (n = 34; 79.1%), consistent with the known epidemiology of high-grade ACL injuries in sport-active populations. Nearly half of the patients (n = 21; 48.8%) were aged 25 years or younger, as shown in Table 1.

**Table 1:** Baseline demographic characteristics (N = 43)

Characteristic	Value (N = 43)
Age (years)	
Mean ± SD	26.9 ± 5.8
Median (range)	25 (19-42)
Sex, n (%)	
Male	34 (79.1%)
Female	9 (20.9%)
Age group, n (%)	
≤25 years	21 (48.8%)
26-30 years	12 (27.9%)
≥31 years	10 (23.3%)

KOOS subscale scores

**Figure 1:**
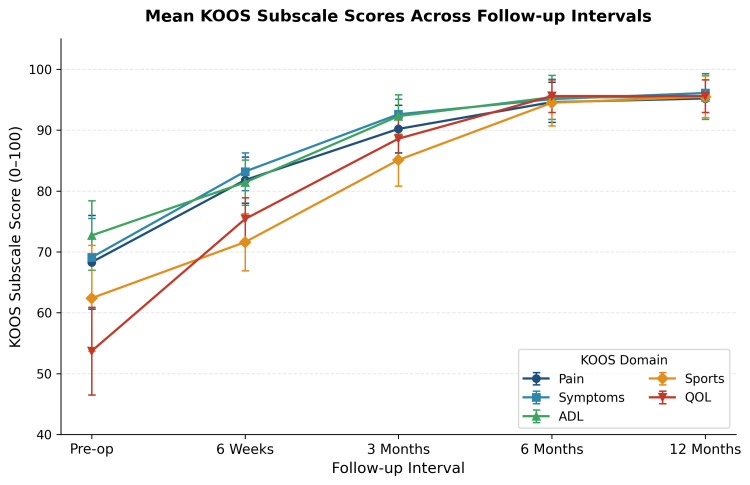
Mean KOOS subscale scores (± SD) across preoperative and follow-up intervals All domains showed statistically significant improvement (p < 0.001). Error bars represent SD. ADL, activities of daily living; KOOS, Knee Injury and Osteoarthritis Outcome Score; QOL, quality of life

All five KOOS domains demonstrated progressive, statistically significant improvement from preoperative baseline through 12-month follow-up, as shown in Table 2 and Figure 1 [17,18]. Friedman’s test yielded p < 0.001 for every domain, with post hoc Wilcoxon signed-rank testing confirming significance at each successive interval.

**Table 2:** Effect size (Cohen’s d) and MCID analysis for KOOS domains: preoperative vs. 12 months (N = 43) Paired Cohen’s d effect sizes for each KOOS domain (preoperative vs. 12-month follow-up), calculated as the mean difference divided by the preoperative SD. All domains demonstrate large effects (d > 3.4). KOOS, Knee Injury and Osteoarthritis Outcome Score; MCID, minimal clinically important difference; QOL, quality of life

KOOS domain	Δ mean (preoperative → 12 months)	Cohen’s d	Interpretation	MCID exceeded?	p-value
Pain	26.9	3.49 (95% CI 2.72-4.26)	Very large	Yes (MCID = 8-10)	<0.001
Symptoms	27	4.22 (95% CI 3.31-5.13)	Very large	Yes (MCID = 8-10)	<0.001
Activities of daily living	22.7	3.98 (95% CI 3.12-4.84)	Very large	Yes (MCID = 8-10)	<0.001
Sports and recreation	33.1	3.80 (95% CI 2.97-4.63)	Very large	Yes (MCID = 8-10)	<0.001
QOL	41.9	5.82 (95% CI 4.60-7.04)	Very large	Yes (MCID = 8-10)	<0.001

The quality-of-life (QOL) subscale recorded the greatest absolute gain, rising from 53.7 ± 7.2 preoperatively to 95.6 ± 2.7 at 12 months, a mean improvement of 41.9 points. KOOS sports and recreation improved by 33.1 points (62.4 ± 8.7 to 95.5 ± 3.4), and KOOS pain improved by 26.9 points (68.3 ± 7.7 to 95.2 ± 3.1). Every domain exceeded the established MCID of 8-10 points [18] by a substantial margin. The progressive narrowing of SDs, from 7.7 preoperatively to 3.1 at 12 months for pain, reflects convergence toward ceiling-level scores and increasing within-cohort homogeneity.

Paired Cohen’s d was calculated for each KOOS domain by dividing the mean preoperative-to-12-month difference by the preoperative SD, as a conservative estimate appropriate for the repeated-measures design [17,18]. All domains yielded large effect sizes, exceeding the conventional large-effect threshold of d ≥ 0.8. The QOL domain had the highest effect size (d = 5.82, 95% CI 4.60-7.04), followed by symptoms (d = 4.22, 95% CI 3.31-5.13), sports and recreation (d = 3.80, 95% CI 2.97-4.63), activities of daily living (d = 3.98, 95% CI 3.12-4.84), and pain (d = 3.49, 95% CI 2.72-4.26). These values represent conservative estimates; the true paired effect sizes may be larger if preoperative and postoperative scores are positively correlated, as is typical in longitudinal rehabilitation studies. These magnitudes confirm that the observed improvements reflect genuine and substantial clinical benefit rather than a statistical artifact.

Lysholm knee score

Preoperatively, no patient achieved a good or excellent Lysholm rating. 46.5% (n = 20) were rated poor and 53.5% (n = 23) fair. Following surgery, the distribution shifted rapidly. By three months, 81.4% (n = 35) had reached a good rating. At six months, 74.4% (n = 32) achieved an excellent score, and by 12 months this proportion rose to 83.7% (n = 36). No patient remained in the poor category beyond six weeks, and the fair category was entirely absent by six months. All between-timepoint comparisons were statistically significant, as shown in Table 3 (p < 0.001).

**Table 3:** Lysholm score category distribution across follow-up intervals (N = 43)

Lysholm category	Pre-op	Six weeks	Three months	Six months	12 months	p-value
Excellent (>81)	0 (0%)	0 (0%)	3 (7.0%)	32 (74.4%)	36 (83.7%)	<0.001
Good (71-80)	0 (0%)	0 (0%)	35 (81.4%)	11 (25.6%)	7 (16.3%)	<0.001
Fair (56-70)	23 (53.5%)	38 (88.4%)	5 (11.6%)	0 (0%)	0 (0%)	<0.001
Poor (<55)	20 (46.5%)	5 (11.6%)	0 (0%)	0 (0%)	0 (0%)	<0.001

IKDC score

IKDC grading confirmed parallel functional recovery [20]. At baseline, 67.4% (n = 29) of patients were classified as Grade D (poor) and 32.6% (n = 14) as Grade C (fair); no patient achieved Grade A (excellent) or Grade B (good). By three months, 74.4% (n = 32) had attained good status. At six months, 83.7% (n = 36) achieved excellent (Grade A), rising to 88.4% (n = 38) at 12 months. No patient remained below good status from six months onward. All comparisons were statistically significant, as shown in Table 4 (p < 0.001).

**Table 4:** IKDC score category distribution across follow-up intervals (N = 43) IKDC, International Knee Documentation Committee

IKDC category	Pre-op	Six weeks	Three months	Six months	12 months	p-value
Excellent (>81)	0 (0%)	0 (0%)	0 (0%)	36 (83.7%)	38 (88.4%)	<0.001
Good (71-80)	0 (0%)	0 (0%)	32 (74.4%)	7 (16.3%)	5 (11.6%)	<0.001
Fair (56-70)	14 (32.6%)	37 (86.0%)	11 (25.6%)	0 (0%)	0 (0%)	<0.001
Poor (<55)	29 (67.4%)	6 (14.0%)	0 (0%)	0 (0%)	0 (0%)	<0.001

Clinical stability: pivot shift and Lachman tests

At both six- and 12-month follow-up, 39 of 43 patients (90.7%) demonstrated Grade 0 (completely negative) pivot shift and Lachman tests. The remaining four patients (9.3%) showed Grade I positivity, indicating mild residual laxity without clinical functional instability. No patient exhibited Grade II or higher laxity at any follow-up assessment, and no patient’s stability grade deteriorated between six and 12 months, confirming the durability of the augmented construct. This outcome is particularly notable given that all enrolled patients presented with Grade III pivot shift preoperatively, the most severe degree of rotational instability.

## Discussion

This prospective cohort study evaluated the functional and clinical outcomes of arthroscopic ACL reconstruction with internal brace (FiberTape) augmentation in 43 patients with Grade III pivot shift, representing the most severe degree of preoperative rotational instability. The results demonstrated consistent and significant improvements across all validated outcome measures over 12 months, with Grade 0 pivot shift observed in 90.7% of patients at final follow-up. The cohort had a mean age of 26.9 years and was 79.1% male, which is consistent with published epidemiological data on sport-related ACL injuries [1,3] and supports the generalizability of these findings to the target population.

The functional improvement across all KOOS domains was substantial [17,18]. Every subscale exceeded the established MCID of 8-10 points [18] by a meaningful margin. The QOL domain alone showed a mean gain of 41.9 points, which is more than four times the minimum threshold. The paired effect sizes (d = 3.49 to 5.82) were considerably larger than those reported in systematic reviews of standard ACL reconstruction, where KOOS improvements of 15-25 points are more common [21,22]. The SDs narrowed over time, from 8.7 before surgery to 3.4 at 12 months in the sports domain. This pattern is consistent with a more homogeneous recovery trajectory than is typically reported after conventional reconstruction, though the absence of a control group precludes direct comparison, and the possibility that ACL reconstruction alone accounts for these gains cannot be excluded.

The Lysholm and IKDC categorical data support these findings [19,20]. After six months, there were no poor or fair ratings, and at 12 months, 83.7% (Lysholm) and 88.4% (IKDC) of patients had achieved excellent status. These results are favorable when considered alongside reported outcomes from conventional hamstring ACL reconstruction in comparable populations. Filbay et al. found that approximately 65-70% of patients reached good-to-excellent outcomes at 12 months after standard reconstruction [21], while Collins et al. noted that meaningful improvements in sports and QOL domains were less consistent without augmentation [22]. While the outcomes observed in the present study are numerically superior, the single-arm design does not permit attribution of these differences to the internal brace specifically. The possibility of patient selection, surgeon experience, or rehabilitation protocol effects contributing to outcomes cannot be excluded. Nonetheless, the rapid categorical improvement observed is consistent with the biomechanical hypothesis that internal brace augmentation provides additional mechanical protection during the ligamentization phase, a period of high biological vulnerability and risk of stretch-induced elongation [23].

The biomechanical rationale for internal brace augmentation is well established. FiberTape acts as a secondary stabilizer that shares tensile load. During early rehabilitation, when the graft is still revascularizing and reorganizing collagen, the FiberTape takes on some of the tensile load that would otherwise be borne solely by the healing tissue. Bachmaier et al. showed that suture tape reinforcement increases construct stiffness and ultimate load-to-failure compared with graft alone, without adverse effects on range of motion when tension is appropriately calibrated [16]. Cook et al. also found that augmented constructs demonstrate better fatigue resistance under repeated loading, which is relevant during early rehabilitation [24]. These biomechanical properties provide a plausible mechanistic basis for the rapid functional gains observed from six weeks onward in this cohort and are consistent with the use of an accelerated rehabilitation protocol, though a causal relationship cannot be established from this study design.

The stability outcomes are noteworthy. Conversion from Grade III preoperative pivot shift to Grade 0 pivot shift was observed in 90.7% of patients at both six and 12 months. Earlier studies of standard ACL reconstruction in high-grade pivot-shift groups have found that 20-35% of patients still demonstrate Grade I or higher laxity at 12 months, particularly in the absence of anterolateral augmentation [9]. Kumar et al. found that internal brace augmentation was associated with reduced residual pivot shift at one year compared with reconstruction alone [25]. Parkes et al. reported lower rates of re-laxity and better rotational stability with suture augmentation in a mixed-grade group [26], and Kitchen et al. described better stability and earlier functional milestones with augmented techniques [27]. The present study adds to this body of evidence by focusing specifically on the Grade III subgroup, where the clinical challenge and potential benefit of supplementary stabilization are greatest, though the design does not permit isolation of the internal brace contribution from that of ACL reconstruction alone. It is important to note that high-grade pivot shift in ACL-deficient knees is a multifactorial phenomenon, often underpinned by concomitant injury to the anterolateral complex (anterolateral ligament and capsule), lateral meniscal pathology, and bony risk factors such as increased posterior tibial slope. In this context, the growing body of evidence supporting lateral extra-articular tenodesis (LET) and anterolateral ligament reconstruction (ALLR) as adjuncts to ACL reconstruction in high-grade pivot-shift patients deserves acknowledgment. Sonnery-Cottet et al. and others have demonstrated that LET directly addresses anterolateral rotational instability and reduces pivot shift more reliably than intra-articular reconstruction alone [9]. While internal brace augmentation targets graft protection rather than direct anterolateral restraint, the favorable stability outcomes observed here raise the question of whether additional benefit might be obtained by combining it with LET or ALLR. Future comparative studies should address this directly. The current study did not routinely assess anterolateral complex integrity on imaging or intraoperatively, which represents a limitation in characterizing the full mechanism underlying the observed stability improvements.

The stable results between six and 12 months, with no patient demonstrating worsening laxity grades, are consistent with durable construct performance over the short term. Graft elongation from repeated loading is a recognized cause of late functional failure in standard reconstruction. The FiberTape’s load-sharing role during early rehabilitation may reduce this risk and contribute to maintenance of rotational stability in the medium term, though this remains hypothetical in the absence of longer follow-up or a comparison group. Whether this protective effect persists beyond 12 months requires investigation in studies with extended follow-up.

There are several limitations to note. First, the absence of a control group undergoing standard ACL reconstruction without augmentation means that the observed improvements cannot be causally attributed to the internal brace; a randomized controlled trial is required to address this definitively. Second, the postoperative exclusion of 22 patients on the basis of intraoperative meniscal findings introduces potential selection bias, as these patients may have had more complex injuries or different baseline characteristics; this is acknowledged as a limitation of the prospective design, and future studies should prespecify stricter enrollment criteria. Third, postoperative stability assessments were performed by the treating surgeon, who was not blinded to the intervention; observer bias in clinical laxity grading cannot therefore be excluded. Fourth, no complications, reoperations, infections, graft failures, or adverse events were recorded during the follow-up period in this cohort, although systematic collection of safety data was not a primary objective of this study and should be reported in future prospective series. Fifth, follow-up was limited to 12 months, precluding assessment of late graft failure, re-rupture rates, osteoarthritis development, and sustained return-to-sport outcomes. Sixth, the anterolateral complex was not routinely evaluated by dedicated MRI sequences or intraoperatively, limiting characterization of the rotational instability mechanism. Finally, excluding patients with significant meniscal injuries, although methodologically appropriate, limits the generalizability of findings to the broader ACL-injured population. Cost and implant availability were not systematically evaluated.

## Conclusions

In this prospective single-arm cohort study, arthroscopic ACL reconstruction with internal brace (FiberTape) augmentation was associated with marked functional and clinical improvement in patients with Grade III preoperative pivot shift over 12 months of follow-up. Statistically significant and clinically meaningful improvements substantially exceeding established MCID thresholds were observed across all three validated outcome measures, such as KOOS, Lysholm, and IKDC, with large paired effect sizes and non-overlapping CIs supporting the robustness of these gains. Conversion from Grade III preoperative pivot shift to Grade 0 pivot shift was observed in 90.7% of patients at both six and 12 months, with no deterioration in laxity between assessments. No patient demonstrated Grade II or higher residual laxity at any follow-up point. These outcomes are consistent with the biomechanical rationale for load-sharing augmentation during early graft rehabilitation. However, as this study lacked a control group, the independent contribution of the internal brace cannot be determined, and causal inference is not warranted. The findings provide preliminary evidence that internal brace augmentation is a clinically feasible adjunct in this high-risk subgroup and support the need for larger, prospective randomized controlled trials with extended follow-up to establish long-term durability and determine whether this technique should be recommended as a standard adjunct in patients with high-grade rotational instability.
